# Type 2 diabetes mellitus and anxiety symptoms: a cross-sectional study in Peru

**DOI:** 10.12688/wellcomeopenres.17328.2

**Published:** 2022-05-13

**Authors:** Guadalupe Arteaga-Zarate, Gina Demarini-Olivares, Paola A. Torres-Slimming, Antonio Bernabe-Ortiz

**Affiliations:** 1 Universidad Peruana de Ciencias Aplicadas, Lima, Peru; 2 CRONICAS Center of Excellence in Chronic Diseases, Universidad Peruana Cayetano Heredia, Lima, Peru; 3 Universidad Científica del Sur, Lima, Peru

**Keywords:** Anxiety, Type 2 diabetes mellitus, Awareness, Peru

## Abstract

Background:

Information about the effect of type 2 diabetes mellitus (T2DM) awareness in the prevalence of anxiety disorders is scarce. Moreover, reports from resource-constrained and semiurban settings are usually focused on hospital-based data, instead of population-based surveys. We aimed to evaluate the association between T2DM and anxiety symptoms, with emphasis on T2DM awareness.

Methods:

A secondary data analysis was conducted using information from a population-based study. The outcome of interest was the presence of anxiety symptoms assessed by the Goldberg anxiety test, while the exposure variable was T2DM, defined using the oral glucose tolerance test. In addition, another definition was used based on self-reported T2DM awareness of previous diagnosis. Prevalence ratios (PR) and 95% confidence intervals (CI) were reported using Poisson regression models.

Results:

Data from 1,607 participants, of mean age 48.2 (SD: 10.6) years, and 809 (50.3%) females, were analyzed. Of all participants, 176 (11.0%; 95% CI: 9.5%–12.6%) had T2DM, 105 (59.7%) were aware of previous diagnosis, and 674 (41.9%; 95% CI: 39.5%–44.4%) had anxiety symptoms. In multivariable model, T2DM was not associated with anxiety symptoms (PR = 1.16; 95% CI: 0.99–1.36); however, individuals aware of T2DM diagnosis had a 36% (95% CI: 14%–64%) greater prevalence of anxiety symptoms compared to those without T2DM. Additionally, those aware of T2DM diagnosis had a 56% (95% CI: 13%-116%) higher probability to have anxiety symptoms compared to those not aware of T2DM diagnosis.

Conclusions:

The association between T2DM and anxiety symptoms was present among those participants who self-reported T2DM diagnosis, as opposed to those with T2DM but not aware and to those without T2DM. Evaluation of anxiety symptoms may be relevant among those with previous T2DM diagnosis.

## Introduction

Anxiety is one of the most frequent psychiatric disorders worldwide ^1^, and is in the top three causes of disability-adjusted life-years (DALYs) among females ^2^. A systematic review of 87 studies from 44 countries estimated that the prevalence of anxiety disorders ranged between 0.9% and 28.3%, whilst past-year prevalence varied between 2.4% and 29.8% ^3^. Moreover, anxiety has been reported to be more prevalent in Latin America, high income regions, and regions with a history of recent conflict ^4^. For example, a population-based survey conducted in Lima, the capital of Peru, reported a prevalence of anxiety disorder between 10% and 15% among adults ^5, 6^.

On the other hand, type 2 diabetes mellitus (T2DM) has been recognized as a major public health concern globally ^7^. Thus, the worldwide prevalence of T2DM has doubled in the last 35 years ^8^. In Peru, the prevalence of T2DM has been estimated to be 7% among adults over 25 years old, whereas this estimate increased to 8.4% in Lima ^9^. Nevertheless, prevalence estimates in northern Peru are higher than national estimates, reaching, on average, a value of 10% ^10^.

Although there is evidence supporting the bidirectional relationship between T2DM and some mental disorders, many of these studies have focused on depression and stress ^11– 13^. Thus, the presence of T2DM may seem to be positively associated with increased depressive symptoms, and similarly, depression may increase the risk of diabetes ^14, 15^, increasing at the same time the risk of complications, morbidity and death ^16^. Nevertheless, a systematic review, using information from 18 studies, reported a 14% prevalence of anxiety disorder among individuals with T2DM compared to 5% among subjects without T2DM ^17^; however, there is scant information about the impact of awareness of a T2DM diagnosis in the prevalence of anxiety disorders. In addition, reports from resource-constrained settings are usually focused on hospital settings, instead of population-based surveys.

As a result, this study aimed to explore the potential influence of the presence of T2DM on having anxiety symptoms, with a particular emphasis of those with T2DM but aware of their diagnosis, using a population-based survey conducted in the north of Peru.

## Methods

### Study design and setting

This study is a secondary analysis of a population-based survey conducted in the semiurban area of Tumbes, a region located in northern Peru, close to the border with Ecuador, between December 2016 and November 2017. Tumbes has an area of approximately 4700 square kilometers and about 245,000 inhabitants ^18^.

### Study participants

Procedures utilized in the population-based study has previously been reported in detail ^19^. A sex-stratified random sampling approach was used. Subjects between 30 and 69 years old, usual residents (≥6 months) of the study area, and able to consent, were invited to participate. Pregnant women, individuals with physical disabilities preventing anthropometric assessment, and those bedridden, were excluded.

### Definition of variables

The outcome of interest in this study was the presence of anxiety symptoms evaluated using the Goldberg Anxiety test. This tool comprises nine items with dichotomic responses (no = 0 and yes = 1 point), and has been validated in Spanish to be used on adults in different countries ^20, 21^, with a sensitivity and specificity of 85% and 65%, respectively. Each positive response adds a point to the total score; the first four items are usually utilized as screening questions, whereas the last five items only ask whether the participant scored two or more points in the first four items. For this study, the nine items were applied to all participants, and a score of ≥4 points was considered as having anxiety symptoms.

The exposure variable was the presence of T2DM, which was defined using the oral glucose tolerance test (OGTT), according to the procedures described by the World Health Organization ^22^. Based on test results, study participants were split into two groups: (1) without type 2 diabetes, those with fasting glucose <126 mg/dL and postprandial glucose <200 mg/dL, and (2) with type 2 diabetes, those with fasting glucose ≥126 mg/dL or postprandial glucose ≥200 mg/dL. For specific sub-analysis, a second definition for the exposure variable was used, in which the type 2 diabetes group was divided into two subgroups depending on self-reported awareness of previous T2DM diagnosis; i.e., whether participants were aware or not of type 2 diabetes diagnosis.

Other variables were also considered in the analysis as potential confounders, including sociodemographic variables, lifestyle behaviors and cardiometabolic factors. Sociodemographic variables included sex (female or male), age (<50 and ≥50 years), education level, collected as years of school accomplished and then divided into three group (<7, 7–11, and ≥12 years), socioeconomic status, evaluated using a wealth index based on family assets and possessions and then split into tertiles, and if the participant was currently working at the moment of the interview (yes or no). Lifestyle behaviors were smoking, defined as the self-report of the consumption of at least one cigarette per day, alcohol disorder, defined using the Alcohol Use Disorder Identification Test (AUDIT) ^23^; physical activity levels were assessed using the International Physical Activity Questionnaire (IPAQ) to estimate the metabolic equivalent of task (MET) ^24^, and split into low levels (<600 MET-minutes/week) and moderate/high levels (those with at least 600 MET-minutes/week). Lastly, cardiometabolic factors considered were body mass index, divided according to traditional cutoffs (<25 Kg/m ^2^, 25 - <30 Kg/m ^2^, and ≥30 Kg/m ^2^), and hypertension status, defined as systolic blood pressure ≥140 mmHg or diastolic blood pressure ≥90 mmHg, or self-report of previous hypertension diagnosis ^25^.

### Study procedures

Questionnaires were administered face-to-face, using tablets with the Open Data Kit (ODK) software. The questionnaire was built using the STEPwise approach to surveillance developed by the World Health Organization (NCD WHO STEPS) ^26^. Information as well as anthropometric assessment were carried out by trained staff.

Regarding OGTT evaluation, individuals were asked to fast for eight to 12 hours before blood sampling. After verifying appropriate fasting period, the first blood sample was drawn, consisting of 7.5 ml of venous blood. After that, participants drank 75 g of anhydrous glucose diluted in 300 ml of water. Two hours later, the second blood sample was taken. During the two-hour period, the questionnaire and the anthropometric measures were performed.

Height (portable stadiometer) and weight (TBF-300A, TANITA Corporation, Tokyo, Japan) were measured using standard procedures. Blood pressure levels were measured in triplicate after a five-minute resting period. Each blood pressure measurement was separated by the other one for at least one minute and were done using an automatic monitor OMRON HEM-780, previously validated for adult populations ^27^.

Blood analyses were carried out by a certified laboratory located in Lima, Peru. Glucose was measured in plasma using a Cobas Modular Platform automated analyzer and reagents were supplied by Roche Diagnostics. Quality control for glucose measurements was provided by Bio-Rad, an independent assessment company.

### Statistical analysis

Analyses were conducted using STATA 16 for Windows (StataCorp, College Station TX, US). Firstly, the characteristics of the study population were described by the exposure and outcome. Categorical variables were described as relative and absolute frequencies, whereas continuous variables were expressed using means and standard deviation (SD). Prevalence and 95% confidence intervals (95% CI) were calculated for variables of interest, and comparisons were carried out using Chi-squared test (two-sided p-values).

To assess the association of interest, crude and adjusted models using Poisson regression with robust variance were created ^28^. In these models, anxiety was considered the dependent variable, whereas the presence of T2DM was the exposure. In addition, a different Poisson regression model was also built to assess the difference on the prevalence of anxiety symptoms comparing those with T2DM but aware to those with T2DM unaware. In all the cases, prevalence ratios (PR) and 95% CI were reported, and collinearity was evaluated utilizing the variance inflation factor (VIF). Finally, because literature is consistent in results about gender as an effect modifier on the potential effect of T2DM on mental health ^29^, we assessed such hypothesis using the likelihood ratio test.

### Ethics

The original study was approved by the IRB at Universidad Peruana Cayetano Heredia, Lima, Peru, and the London School of Hygiene and Tropical Medicine, London, UK. Written informed consent was obtained from participants. This analysis was approved by the ethical committee of the Universidad Peruana de Ciencias Aplicadas, Lima, Peru. The database is available in Figshare ^30^, and did not contain identifying information, to guarantee appropriate anonymity and confidentiality.

## Results

### Characteristics of the study population

A total of 1,607 participant responses were analyzed, including 809 (50.3%) females, with a mean age of 48.2 (SD: 10.6) years; 518 (32.2%) had six or less years of education. Of all participants, 176 (11.0%; 95% CI: 9.5% - 12.6%) had T2DM, and 105 (59.7%) of them were aware of a previous diagnosis, with an average of 6.3 (SD: 6.1) years since diagnosis. Older age, low education, currently working, alcohol disorder, low physical activity levels, high body mass index, and hypertension were variables associated with having T2DM ( Table 1).

**Table 1.** Characteristics of the study population by type 2 diabetes mellitus (T2DM) status.

	Type 2 diabetes status			
	No T2DM	T2DM unaware	T2DM aware	p-value
	(n = 1431)	(n = 71)	(n = 105)	
***Sex***				
Male	724 (50.6%)	29 (40.8%)	45 (42.9%)	0.10
Female	707 (49.4%)	42 (59.2%)	60 (57.1%)	
***Age***				
< 50 years	861 (60.2%)	36 (50.7%)	23 (21.9%)	< 0.001
50+ years	570 (39.8%)	35 (49.3%)	82 (78.1%)	
***Education level***				
< 7 years	438 (30.6%)	27 (38.0%)	53 (50.5%)	< 0.001
7 – 11 years	675 (47.2%)	32 (45.1%)	41 (39.0%)	
12+ years	318 (22.2%)	12 (16.9%)	11 (10.5%)	
***Socioeconomic level***				
Low	470 (32.8%)	25 (35.2%)	43 (41.0%)	0.34
Middle	497 (34.7%)	20 (28.2%)	33 (31.4%)	
High	464 (32.4%)	26 (36.6%)	29 (27.6%)	
***Currently working***				
Yes	992 (69.3%)	42 (59.2%)	56 (53.3%)	< 0.001
***Daily smoking***				
Yes	84 (5.9%)	2 (2.8%)	6 (5.7%)	0.56
***Alcohol disorder***				
Yes	113 (7.9%)	8 (11.3%)	0 (0.0%)	0.006
***Physical activity***				
Low levels	522 (36.5%)	27 (38.0%)	55 (52.4%)	0.005
***Body mass index***				
Normal	388 (27.1%)	11 (15.5%)	26 (24.8%)	0.02
Overweight	625 (43.7%)	28 (39.4%)	53 (50.5%)	
Obesity	418 (29.2%)	32 (45.1%)	26 (24.7%)	
***Hypertension***				
Yes	345 (24.1%)	25 (35.2%)	47 (44.8%)	< 0.001

### Anxiety symptoms and associated factors

Overall, 674 (41.9%; 95% CI: 39.5% - 44.4%) individuals had anxiety symptoms. These were more frequent among females (56.0% against 27.7%) than males, and among those not working (53.4% against 36.5%) compared to those currently working ( Table 2). In addition, daily smoking, alcohol disorder, physical activity and body mass index were behavioral variables associated with the presence of anxiety symptoms.

**Table 2.** Characteristics of the study population by anxiety symptoms.

	Anxiety symptoms		
	No (n = 933)	Yes (n = 674)	p-value
***Sex***			
Male	577 (61.8%)	221 (32.8%)	< 0.001
Female	356 (38.2%)	453 (67.2%)	
***Age***			
< 50 years	540 (57.9%)	380 (56.4%)	0.55
50+ years	393 (42.1%)	294 (43.6%)	
***Education level***			
< 7 years	295 (31.6%)	223 (33.1%)	0.55
7 – 11 years	445 (47.7%)	303 (44.9%)	
12+ years	193 (20.7%)	148 (22.0%)	
***Socioeconomic level***			
Low	306 (32.8%)	232 (34.4%)	0.68
Middle	318 (34.1%)	232 (34.4%)	
High	309 (33.1%)	210 (31.2%	
***Currently working***			
No	241 (25.8%)	276 (40.9%)	< 0.001
Yes	692 (74.2%)	398 (59.1%)	
***Daily smoking***			
No	868 (93.0%)	647 (96.0%)	0.01
Yes	65 (7.0%)	27 (4.0%)	
***Alcohol disorder***			
No	847 (90.8%)	639 (94.8%)	0.003
Yes	86 (9.2%)	35 (5.2%)	
***Physical activity***			
Moderate/high levels	613 (65.7%)	390 (57.9%)	0.001
Low levels	320 (34.3%)	284 (42.1%)	
***Body mass index***			
Normal	265 (28.4%)	160 (23.7%)	0.03
Overweight	413 (44.3%)	293 (43.5%)	
Obesity	255 (27.3%)	221 (32.8%)	
***Hypertension***			
No	704 (75.5%)	486 (72.1%)	0.13
Yes	229 (24.5%)	188 (27.9%)	

### Association between type 2 diabetes and anxiety symptoms

In the multivariable model, and after controlling for different sociodemographic and behavioral factors, T2DM was not associated with the presence of anxiety symptoms (PR = 1.16; 95% CI: 0.99 – 1.36). Nevertheless, those individuals with T2DM, but aware of their diagnosis had a 36% (95% CI: 14% – 64%) greater prevalence of anxiety symptoms compared to those without T2DM ( Table 3). Moreover, those aware of their T2DM diagnosis had a 56% (95% CI: 13% – 116%) higher probability to have anxiety symptoms compared to those not aware of their T2DM diagnosis. Finally, gender was not an effect modifier of the association of interest (p=0.48).

**Table 3.** Effect of type 2 diabetes mellitus (T2DM) on the presence of anxiety symptoms: crude and adjusted models.

	Anxiety symptoms		Crude model	Adjusted model *
	No (n = 933)	Yes (n = 674)	PR (95% CI)	PR (95% CI)
***T2DM***				
No T2DM	846 (59.1%)	585 (40.9%)	1 (Reference)	1 (Reference)
With T2DM	87 (49.4%)	89 (50.6%)	**1.24 (1.06 – 1.45)**	1.16 (0.99 – 1.36)
***T2DM and awareness***				
No T2DM	846 (59.1%)	585 (40.9%)	1 (Reference)	1 (Reference)
With T2DM, but unaware	44 (62.0%)	27 (38.0%)	0.93 (0.69 – 1.26)	0.87 (0.66 – 1.16)
With T2DM, but aware	43 (41.0%)	62 (59.0%)	**1.44 (1.22 – 1.71)**	**1.37 (1.14 – 1.64)**

* Adjusted for sex, age, education level, socioeconomic status, daily smoking, alcohol disorder, and physical activity

## Discussion

### Main findings

According to our results, there was no association between T2DM and the presence of anxiety symptoms at the population level; however, in our multivariable model, those individuals aware of their T2DM diagnosis had, on average, 37% and 57% greater prevalence of anxiety symptoms compared to those without T2DM and those not aware of T2DM diagnosis, respectively. Additionally, more than 40% of individuals from the general population had symptoms of anxiety and more than one in 10 had T2DM.

### Comparison with previous studies

Several studies have assessed the relationship between T2DM and anxiety. For example, a systematic review of 12 studies reported a significant positive association between T2DM and anxiety disorder and elevated anxiety symptoms assessing data from 12,626 individuals ^14^. Nevertheless, a more recent review did not find a longitudinal association between baseline T2DM and incident anxiety, but instead an association between baseline anxiety and incident T2DM ^31^.

Different cross-sectional studies have reported the association between T2DM and anxiety. Thus, a nationwide survey in Taiwan reported that the prevalence of anxiety disorders was higher among patients with T2DM than those in the general population ^32^. In addition, a study conducted in Ireland showed that anxiety symptoms were considerably higher among cases of T2DM; nevertheless, the sample was enrolled from hospital/general practitioner shared care instead of the general population ^33^. In a study conducted in medical centers in Brazil found that some psychiatric disorders, i.e., generalized anxiety disorder, phobic-anxious disorder and mood disorders, were more frequent among those with T2DM than those without the condition ^34^.

Our results agree with these previous reports, but expand on showing that much of the association between T2DM and anxiety symptoms in a resource-constrained setting, is related to the awareness of that chronic condition. A study conducted in The Netherlands using a population-based cohort of 90,686 participants found that both diagnosed and undiagnosed T2DM were associated with the presence of anxiety disorders; however, the odds of experiencing anxiety were significantly higher among diagnosed (i.e., aware) compared with undiagnosed (i.e., unaware) cases ^35^.

### Public health relevance

Our findings support the concept that awareness of T2DM explains the higher prevalence of anxiety symptoms among individuals with this condition. This association, especially among those aware of their T2DM diagnosis, may be related to having lived with this chronic condition and diabetes distress for longer ^36^. In addition, the need for continuous monitoring, taking antidiabetic medication, and the increased risk for future complications or other T2DM-related morbidities may induce anxiety among individuals with T2DM ^16, 37^.

Some chronic conditions have been associated with mental health problems, depending on the time of diagnosis. Hypertension, for example, has been associated with depressive symptoms, especially in the first years after diagnosis ^38^. It is therefore necessary to guarantee appropriate mental health assessments of participants with noncommunicable conditions, especially common ones such as hypertension or T2DM. Different tools are available to assess mental health, including the Patient Health Questionnaire 9 (PHQ-9) for depression, and the Goldberg Anxiety test, with nine items, or the General Anxiety Disorder 7 (GAD-7) for anxiety. These tools are short, and can be easily implementable and used during clinical attention to appropriately detect non-communicable disease cases with mental health problems that required adequate management.

### Strength and limitations

The present analysis was conducted using a population-based survey conducted in an area with a high prevalence of T2DM. Cases with diabetes were detected using the OGTT, gold standard for T2DM diagnosis, and a valid tool for anxiety was utilized. Nevertheless, there are limitations that should be highlighted. First, because of the cross-sectional nature of the study, only associations can be reported. Although literature has shown bidirectional association between T2DM and mental health problems such as depression due to hypothalamic-pituitary-adrenal axis dysregulation ^39^, such relationship is controversial in the case of stress and anxiety. Second, some selection bias may have been introduced as the study sample was recruited in a setting with high prevalence of T2DM. Third, some recall bias may arise, especially for covariates such as smoking, alcohol disorder and physical activity. Finally, some variables, such a previous history of, or treatment for, anxiety, as well as potential confounders, including, but not limited to, comorbidities or T2DM complications, were not assessed.

## Conclusions

The association between T2DM and anxiety symptoms was only present among those aware of T2DM diagnosis, but not among those unaware. Evaluation and follow-up of anxiety symptoms may be relevant among those with previous T2DM diagnosis.

## Data availability

### Underlying data

Figshare:Bernabe-Ortiz, Antonio (2021): T2DM and anxiety,

https://doi.org/10.6084/m9.figshare.16862191.v2 ^30^

This project contains the following underlying data:

- T2DM and anxiety v11.csv (dataset)
- Dictionary (110521).txt (key to variable abbreviations)

Data are available under the terms of the Creative Commons Attribution 4.0 International license (CC-BY 4.0).
