# Supplementary material for: Type 2 diabetes mellitus and anxiety symptoms: a cross-sectional study in Peru
Source: Wellcome Open Res. 2022 May 13;6:331. Originally published 2021 Dec 6. [Version 2] doi: 10.12688/wellcomeopenres.17328.2 (PMC9111365; doi:10.12688/wellcomeopenres.17328.2)
Supplement: Peer review history

# Peer review: Reviewer response for version 2

**DOI:** 10.21956/wellcomeopenres.19823.r50528

**Published:** 2022-05-16

**Authors:** Rosane Harter Griep

**Affiliations:** 1 Laboratório de Educação em Ambiente e Saúde, Instituto Oswaldo Cruz, Fundação Oswaldo Cruz, Rio de Janeiro, Brazil

I am satisfied with the improvements made by the authors.

Is the work clearly and accurately presented and does it cite the current literature?

Yes

If applicable, is the statistical analysis and its interpretation appropriate?

Yes

Are all the source data underlying the results available to ensure full reproducibility?

Yes

Is the study design appropriate and is the work technically sound?

Yes

Are the conclusions drawn adequately supported by the results?

Yes

Are sufficient details of methods and analysis provided to allow replication by others?

Yes

Reviewer Expertise:

Public health, occupational health, cardiovascular diseases

I confirm that I have read this submission and believe that I have an appropriate level of expertise to confirm that it is of an acceptable scientific standard.

# Peer review: Reviewer response for version 2

**DOI:** 10.21956/wellcomeopenres.19823.r50527

**Published:** 2022-05-16

**Authors:** Pilar Serrano-Gallardo

**Affiliations:** 1 Nursing Department, School of Medicine, IDIPHISA, INAECU, Autonomous University of Madrid, Madrid, Spain

No further comments.

Is the work clearly and accurately presented and does it cite the current literature?

Yes

If applicable, is the statistical analysis and its interpretation appropriate?

Partly

Are all the source data underlying the results available to ensure full reproducibility?

Yes

Is the study design appropriate and is the work technically sound?

Yes

Are the conclusions drawn adequately supported by the results?

Partly

Are sufficient details of methods and analysis provided to allow replication by others?

Yes

Reviewer Expertise:

Public Health, Chronic diseases, Social Health Inequalities

I confirm that I have read this submission and believe that I have an appropriate level of expertise to confirm that it is of an acceptable scientific standard.

# Peer review: Reviewer response for version 1

**DOI:** 10.21956/wellcomeopenres.19158.r50192

**Published:** 2022-05-09

**Authors:** Pilar Serrano-Gallardo

**Affiliations:** 1 Nursing Department, School of Medicine, IDIPHISA, INAECU, Autonomous University of Madrid, Madrid, Spain

- The topic is of interest because both T2DM and anxiety are important public health problems with a major impact on people's health, as well as on health systems and at the societal level. Moreover, the relationship between the two problems remains a phenomenon that needs to be investigated to shed light on the causal pathways between anxiety and other mental health problems and T2DM. Precisely for this reason, the Introduction lacks a reflection on this issue, which would be necessary. The authors reflect that the presence of T2DM seems to be positively associated with an increase in depressive and anxiety symptoms (citation 12), suggesting that T2DM could be a risk factor for triggering depressive and anxiety symptoms. However, this temporal sequence is not clear in the literature, as are its pathogenic mechanisms, see for example the systematic review by Rotella and Mannuci ^1^ who identified an increased risk of developing diabetes in depressed versus non-depressed subjects, as well as in those taking antidepressant drugs and those with untreated depression. 
- Although the method section states that the exposure variable is T2DM, the aim of the study should clarify whether the intention is to explore the influence of the presence of T2DM on having anxiety symptoms or vice versa. Also, within the Method, the statistical analysis should reflect which variable has been taken as the dependent variable for the regression model.
- In the same sense as mentioned in the previous point, table 3 should have a clearer title. In addition, and in relation to the results described in this table in the text, it would be more appropriate to include the PR and their 95%CI for the category "With T2DM, but aware". In the same paragraph, it is not clear where the data is derived from in the sentence: "In addition, those who were aware of their T2DM diagnosis were 56% (95% CI: 13% - 116%) more likely to have anxiety symptoms compared to those who were unaware of their T2DM diagnosis".
- The main limitation of the study is the cross-sectional nature of the design, which prevents capturing the temporal sequence, which is essential in the causal analysis. Although this is reflected in the Discussion, further explanation should be given in this section to support the authors' approach that relies on T2DM as an exposure variable for anxiety (see Joseph et al ^2^). 

Is the work clearly and accurately presented and does it cite the current literature?

Yes

If applicable, is the statistical analysis and its interpretation appropriate?

Partly

Are all the source data underlying the results available to ensure full reproducibility?

Yes

Is the study design appropriate and is the work technically sound?

Yes

Are the conclusions drawn adequately supported by the results?

Partly

Are sufficient details of methods and analysis provided to allow replication by others?

Yes

Reviewer Expertise:

Public Health, Chronic diseases, Social Health Inequalities

I confirm that I have read this submission and believe that I have an appropriate level of expertise to confirm that it is of an acceptable scientific standard, however I have significant reservations, as outlined above.

## References

[1] : Depression as a risk factor for diabetes: a meta-analysis of longitudinal studies. *J Clin Psychiatry* .2013;74(1) : 10.4088/JCP.12r07922 31-7 10.4088/JCP.12r07922 23419223

[2] : Cortisol dysregulation: the bidirectional link between stress, depression, and type 2 diabetes mellitus. *Ann N Y Acad Sci* .1391(1) : 10.1111/nyas.13217 20-34 10.1111/nyas.13217 27750377PMC5334212

# Response

**Published:** 2022-05-10

**Authors:** Antonio Bernabe-Ortiz

**Affiliations:** Universidad Peruana Cayetano Heredia, Peru

**The topic is of interest because both T2DM and anxiety are important public health problems with a major impact on people's health, as well as on health systems and at the societal level. Moreover, the relationship between the two problems remains a phenomenon that needs to be investigated to shed light on the causal pathways between anxiety and other mental health problems and T2DM. Precisely for this reason, the Introduction lacks a reflection on this issue, which would be necessary. The authors reflect that the presence of T2DM seems to be positively associated with an increase in depressive and anxiety symptoms (citation 12), suggesting that T2DM could be a risk factor for triggering depressive and anxiety symptoms. However, this temporal sequence is not clear in the literature, as are its pathogenic mechanisms, see for example the systematic review by Rotella and Mannuci** **^1^** **who identified an increased risk of developing diabetes in depressed versus non-depressed subjects, as well as in those taking antidepressant drugs and those with untreated depression.**

***Response:*** We have changed the third paragraph of the Introduction to include this topic. Now it reads: “ *Although there is evidence supporting the bidirectional relationship between T2DM and some mental health disorders, many of these studies have focused on depression and stress. Thus, the presence of T2DM may seem to be positively associated with increased depressive symptoms, and similarly, depression may increase the risk of diabetes* *^11^* *^,^* *^12^* *,* *increasing at the same time the risk of complications, morbidity and death* *^13^*.”

**Although the method section states that the exposure variable is T2DM, the aim of the study should clarify whether the intention is to explore the influence of the presence of T2DM on having anxiety symptoms or vice versa. Also, within the Method, the statistical analysis should reflect which variable has been taken as the dependent variable for the regression model.**

***Response:*** We have modified the aim of the study as suggested by the reviewer. Now it reads: “… *this study aimed to explore the potential influence of the presence of T2DM on having anxiety symptoms, with a particular emphasis on those aware of a previous T2DM diagnosis*…”

 We have also clarified this in the *Statistical Analysis* section: “ *In these models, anxiety was considered the dependent variable, whereas the presence of T2DM was the exposure.*”

**In the same sense as mentioned in the previous point, table 3 should have a clearer title. In addition, and in relation to the results described in this table in the text, it would be more appropriate to include the PR and their 95%CI for the category "With T2DM, but aware". In the same paragraph, it is not clear where the data is derived from in the sentence: "In addition, those who were aware of their T2DM diagnosis were 56% (95% CI: 13% - 116%) more likely to have anxiety symptoms compared to those who were unaware of their T2DM diagnosis".**

***Response:*** We have changed the title of Table 3. Now it reads: “ *Effect of type 2 diabetes mellitus (T2DM) on the presence of anxiety symptoms: crude and adjusted models*”.

 In addition, we have added the PR and 95% CI as suggested: “ *those individuals with T2DM, but aware of their diagnosis had a 37% (95% CI: 14% – 64%) greater prevalence of anxiety symptoms compared to those without T2DM*”.

 Regarding the sentence: “In addition, those who were aware of their T2DM diagnosis were 56% (95% CI: 13% - 116%) more likely to have anxiety symptoms compared to those who were unaware of their T2DM diagnosis”, we compared those with T2DM but aware to those with T2DM but unaware. So, the result is not in the table but instead in text only. This point is important because those unaware should be similar to those without T2DM. We have clarified that point in the *Statistical Analysis* section as this comparison was not included: “ *In addition, a different Poisson regression model was also built to assess the difference in the prevalence of anxiety symptoms comparing those with T2DM but aware to those with T2DM unaware*”.

**The main limitation of the study is the cross-sectional nature of the design, which prevents capturing the temporal sequence, which is essential in the causal analysis. Although this is reflected in the Discussion, further explanation should be given in this section to support the authors' approach that relies on T2DM as an exposure variable for anxiety (see Joseph et al** **^2^** **).**

***Response:*** We have added some lines regarding this topic according to the reviewer's suggestion: “First, because of the cross-sectional nature of the study, only associations can be reported. Although literature has shown bidirectional association between T2DM and mental health problems such as depression due to hypothalamic-pituitary-adrenal axis dysregulation, such relationship is controversial in the case of stress and anxiety.”

# Peer review: Reviewer response for version 1

**DOI:** 10.21956/wellcomeopenres.19158.r48941

**Published:** 2022-05-03

**Authors:** Rosane Harter Griep

**Affiliations:** 1 Laboratório de Educação em Ambiente e Saúde, Instituto Oswaldo Cruz, Fundação Oswaldo Cruz, Rio de Janeiro, Brazil

Thank you for the opportunity to review this interesting article. The article aimed to evaluate the association between type 2 diabetes mellitus (T2DM) and anxiety symptoms, with an emphasis on T2DM awareness. According to the authors, the information about the effect of T2DM awareness on the prevalence of anxiety disorders is scarce, especially in population-based studies. So, the analyses of the article are based on a secondary data analysis using information from a population-based study.

- Despite the relevance of the topic, my main concern with the article is related to the cross-sectional design for analyses. Some studies have shown that mental health could be a risk factor for diabetes. On the other side, some studies have indicated high rates of co-occurrence of the two diseases and support that the relationships between diabetes and depression are bidirectional (please see Zhuang *et al*., 2017 ^1^, van der Feltz-Cornelis *et al*., 2021 ^2^). Please include this perspective in the introduction and justify the option of the association between DM to anxiety symptoms. I also suggest testing the bi-directionality of the association between mental health and diabetes.
- Please consider including a recently published article about the same subject as the presented article, among the Latin American population (Barbosa *et al*., 2022). The results are based on a relevant Brazilian cohort study. The article showed results by gender and observed that women classified with DM were at 54% greater risk (95% CI = 1.06-2.19) of depressive episodes compared to women classified as non-DM. No significant associations were observed for men. I'd like to suggest the authors evaluate gender as a modifier effect on the analyses. The literature is consistent in results about gender differences in the mental health issues (mental health is usually worse among women compared to men), and also the probability that T2DM awareness is different by gender (women presented higher chances of T2DM awareness). So, gender could be an important effect modifier variable.
- Have you collected the duration of T2DM diagnosis? Maybe the time of knowledge of diabetes could be an influencer on the association (more recent diagnosis, worse mental health).
- I am not sure if lifestyle behaviors could be considered potential confounders. For example, physical activities could be considered a mediator between T2DM and mental health (especially among those with a previous diagnosis of T2DM).
- The discussion section is well written.

Is the work clearly and accurately presented and does it cite the current literature?

Yes

If applicable, is the statistical analysis and its interpretation appropriate?

Yes

Are all the source data underlying the results available to ensure full reproducibility?

Yes

Is the study design appropriate and is the work technically sound?

Yes

Are the conclusions drawn adequately supported by the results?

Yes

Are sufficient details of methods and analysis provided to allow replication by others?

Yes

Reviewer Expertise:

Public health, occupational health, cardiovascular diseases

I confirm that I have read this submission and believe that I have an appropriate level of expertise to confirm that it is of an acceptable scientific standard, however I have significant reservations, as outlined above.

## References

[1] : Quantitative assessment of the bidirectional relationships between diabetes and depression. *Oncotarget* .2017;8(14) : 10.18632/oncotarget.15051 23389-23400 10.18632/oncotarget.15051 28177893PMC5410312

[2] : Treatment for comorbid depressive disorder or subthreshold depression in diabetes mellitus: Systematic review and meta-analysis. *Brain Behav* .11(2) : 10.1002/brb3.1981 e01981 10.1002/brb3.1981 33274609PMC7882189

[3] : The association between diabetes mellitus and incidence of depressive episodes is different based on sex: insights from ELSA-Brasil. *Therapeutic Advances in Endocrinology and Metabolism* .2022;13: 10.1177/20420188221093212 10.1177/20420188221093212 PMC901938235464879

# Response

**Published:** 2022-05-10

**Authors:** Antonio Bernabe-Ortiz

**Affiliations:** Universidad Peruana Cayetano Heredia, Peru

**Despite the relevance of the topic, my main concern with the article is related to the cross-sectional design for analyses. Some studies have shown that mental health could be a risk factor for diabetes. On the other side, some studies have indicated high rates of co-occurrence of the two diseases and support that the relationships between diabetes and depression are bidirectional (please see Zhuang *et al*., 2017** **^1^** **, van der Feltz-Cornelis *et al*., 2021** **^2^** **). Please include this perspective in the introduction and justify the option of the association between DM to anxiety symptoms. I also suggest testing the bi-directionality of the association between mental health and diabetes.**

***Response: *** We have rephrased the third paragraph of the Introduction to include this topic and the references provided. Nevertheless, none of the references given by the reviewer studied the bidirectional association between T2DM and anxiety symptoms. Now it reads: “ *Although there is evidence supporting the bidirectional relationship between T2DM and some mental health disorders, many of these studies have focused on depression and stress. Thus, the presence of T2DM may seem to be positively associated with increased depressive symptoms, and similarly, depression may increase the risk of diabetes…*”.

 We have decided not to test the bidirectionality of the association between T2DM and anxiety as temporality is not evaluable in cross-sectional studies. Anxiety was evaluated in the last 2 weeks prior to the application of the questionnaire, and as a result, anxiety cannot happen before type 2 diabetes.

**Please consider including a recently published article about the same subject as the presented article, among the Latin American population (Barbosa *et al*., 2022). The results are based on a relevant Brazilian cohort study. The article showed results by gender and observed that women classified with DM were at 54% greater risk (95% CI = 1.06-2.19) of depressive episodes compared to women classified as non-DM. No significant associations were observed for men. I'd like to suggest the authors evaluate gender as a modifier effect on the analyses. The literature is consistent in results about gender differences in the mental health issues (mental health is usually worse among women compared to men), and also the probability that T2DM awareness is different by gender (women presented higher chances of T2DM awareness). So, gender could be an important effect modifier variable.**

***Response: *** We have added the reference as requested. On the other hand, we assessed the potential effect of modification of gender on the relationship between T2DM and anxiety. Our results were not significant, and as a result, we decided not to add such results. Based on the comment of the reviewer, we have added such finding in the Methods and Results section.

 In the Methods, Statistical analysis section, we add: “Finally, because literature is consistent in results about gender as an effect modifier on the potential effect of T2DM on mental health, we assessed such hypothesis using the likelihood ratio test.”

 Similarly, we added the finding in the Results section: “Finally, gender was not an effect modifier of the association of interest (p=0.48).”

**Have you collected the duration of T2DM diagnosis? Maybe the time of knowledge of diabetes could be an influencer on the association (more recent diagnosis, worse mental health).**

***Response: *** Unfortunately, that information was not collected, as the original study was centered on recruited participants with recent T2DM diagnoses (those unaware).

**I am not sure if lifestyle behaviors could be considered potential confounders. For example, physical activities could be considered a mediator between T2DM and mental health (especially among those with a previous diagnosis of T2DM).**

***Response: *** This is a good point highlighted by the reviewer. We decided to include physical activity as a potential confounder as this variable is related to mental health outcomes. For example, high levels of physical activity are associated with a lower risk of depression or stress and potentially could be associated with anxiety. Similarly, evidence suggests that smoking and alcohol disorders are associated with mental health outcomes.

**The discussion section is well written.**

***Response:*** Thanks.
